# The role of vitamin D deficiency on COVID-19: a systematic review and meta-analysis of observational studies

**DOI:** 10.4178/epih.e2021074

**Published:** 2021-09-23

**Authors:** Mehmet Onur Kaya, Esra Pamukçu, Burkay Yakar

**Affiliations:** 1 Department of Biostatistics and Medical Informatics, Firat University School of Medicine, Elazığ, Turkey; 2 Department of Statistics, Faculty of Science, Fırat University, Elazığ, Turkey; 3 Department of Family Medicine, Firat University School of Medicine, Elazığ, Turkey

**Keywords:** Vitamin D deficiency, COVID-19, SARS-CoV-2, Meta-analysis, Systematic review

## Abstract

**OBJECTIVES:**

Although vaccination has started, coronavirus disease 2019 (COVID-19) poses a continuing threat to public health. Therefore, in addition to vaccination, the use of supplements to support the immune system may be important. The purpose of this study was to synthesize evidence on the possible effect of low serum vitamin D levels (25[OH]D<20 ng/mL or 50 nmol/L) on COVID-19 infection and outcomes.

**METHODS:**

We searched Google Scholar, PubMed, Scopus, Web of Science, and ScienceDirect without any language restrictions for articles published between January 1 and December 15, 2020. We performed 3 meta-analyses (called vitamin D and COVID-19 infection meta-analysis [D-CIMA], vitamin D and COVID-19 severity meta-analysis [D-CSMA], and vitamin D and COV ID-19 mortality meta-analysis [D-CMMA] for COVID-19 infection, severity, and mortality, respectively) to combine odds ratio values according to laboratory measurement units for vitamin D and the measured serum 25(OH)D level.

**RESULTS:**

Twenty-one eligible studies were found to be relevant to the relationship between vitamin D and COVID-19 infection/outcomes (n=205,869). The D-CIMA meta-analysis showed that individuals with low serum vitamin D levels were 1.64 times (95% confidence interval [CI], 1.32 to 2.04; p<0.001) more likely to contract COVID-19. The D-CSMA meta-analysis showed that people with serum 25(OH)D levels below 20 ng/mL or 50 nmol/L were 2.42 times (95% CI, 1.13 to 5.18; p=0.022) more likely to have severe COVID-19. The D-CMMA meta-analysis showed that low vitamin D levels had no effect on COVID-19 mortality (OR, 1.64; 95% CI, 0.53 to 5.06, p=0.390).

**CONCLUSIONS:**

According to our results, vitamin D deficiency may increase the risk of COVID-19 infection and the likelihood of severe disease. Therefore, we recommend vitamin D supplementation to prevent COVID-19 and its negative outcomes.

## INTRODUCTION

Although coronavirus disease 2019 (COVID-19) is asymptomatic or has mild symptoms in the majority of the population, it may lead to death by causing serious clinical syndromes such as pneumonia, acute respiratory distress syndrome (ARDS), myocarditis, microvascular thrombosis, and cytokine storm in some patients [1]. Therefore, there is a need to protect individuals from COVID-19, which is reported to have become more contagious as it has mutated, and to reduce the risk of severe disease and, consequently, the mortality rate. The COVID-19 vaccines that have been approved for emergency use have been a gleam of hope in the global struggle against COVID-19. However, the effects of vaccines on the immune system have not been clearly proven, and there have been some cases of severe COVID-19 cases, and even deaths, in patients who were fully vaccinated. As we have learned from similar viral infections, supplements such as vitamin D to support the immune system play an important role in addition to vaccination.

Extensive research has explored the effects of vitamin D on the treatment and complications of COVID-19 and its potential contribution to the reduction of COVID-19 incidence. Vitamin D exerts antiviral activity and inhibits viral replication by stimulating the release of cathelicidin and defensin proteins in monocytes and macrophages [2,3]. Vitamin D plays an important role in preventing respiratory system infections due to its effects such as stimulating the chemotaxis of T-lymphocytes and clearing respiratory pathogens by inducing apoptosis and autophagy in the infected epithelium [4]. It has been reported that low T-lymphocyte levels were found in some COVID-19 patients with severe symptoms [5]. Since vitamin D supplementation increases the level of T-lymphocytes [6], this finding provides support for the hypothesis that vitamin D could be useful in the treatment of COVID-19.

The severe progression of COVID-19 in some patients is one of the most important problems of the pandemic. Studies have pointed out an increased rate of thrombotic events and cytokine storm in severe COVID-19 patients. These events are responsible for fatal outcomes [7-9]. It is well known that vitamin D sufficiency reduces the risk of cytokine storm and regulates thrombotic pathways [10,11]. It has been reported that vitamin D sufficiency may attenuate the increased levels of inflammatory markers and cytokine storm during COVID-19 disease and that vitamin D deficiency (VDD) may be related to COVID-19 severity and mortality [12,13]. Consequently, the effect of VDD on COVID-19 infection and outcomes is topic that has attracted considerable interest.

Most current studies about this subject have focused on the effect of VDD on COVID-19 infection, severity, and treatment, and findings on the relationship between VDD and COVID-19 mortality are limited. Therefore, as reported in the literature [13-15], more comprehensive clinical studies are still needed.

Three meta-analyses of this issue have been published, one of which is a preprint article [16-18]. In these studies, we identified some problems such as inconsistent definitions of VDD (e.g., 25[OH]D < 20 or 12 ng/mL, < 50 or 30 nmol/L), the combination of different summary statistics (e.g., odds ratio [OR], risk ratio [RR], and hazard ratio [HR]), and a tendency to perform a meta-analysis of an individual study and give the results as if they were pooled results of a meta-analysis. In the Endocrine Society’s Practice Guidelines on Vitamin D, VDD is defined as a 25(OH)D level <20 ng/mL or 50 nmol/L, vitamin D insufficiency as 21-29 ng/mL, and vitamin D sufficiency as at least 30 ng/mL for maximum musculoskeletal health [19]. This is the first systematic review and meta-analysis that establishes the association between COVID-19 infection/outcomes and VDD according to the common cut-off value (defining VDD as a 25[OH]D level < 20 ng/mL or 50 nmol/L) proposed by advisory bodies. The purpose of this study was to synthesize evidence on the associations of VDD with COVID-19 infection, severity, and mortality and to provide an analytical contribution to the literature on the role of vitamin D supplementation in treatment and prevention protocols for COVID-19.

## MATERIALS AND METHODS

Throughout this systematic review and meta-analysis study, we followed the PRISMA (Preferred Reporting Items for Systematic Reviews and Meta-Analyses) guidelines [20] (Supplementary Material 1).

### Search strategy

We searched Google Scholar, PubMed, Scopus, Web of Science, and ScienceDirect without any language restriction and publication status limit. Our search keywords were “vitamin D” and “COVID-19,” “vitamin D” and “SARS-CoV-2,” and “vitamin D” and “coronavirus disease.” Articles that included the search keywords in their title and were published between January 1 and December 15, 2020 were chosen (Supplementary Material 2). We also screened the reference lists of other meta-analysis studies. Two independent researchers (MOK and EP) screened the titles, abstracts, and full-texts for inclusion in qualitative and quantitative analyses.

### Selection criteria

The inclusion criteria for eligible studies were as follows: (1) cohort or case-control studies on the association between VDD and COVID-19 disease; (2) studies defining VDD according to the common definition (25[OH]D<20 ng/mL or 50 nmol/L) as the exposure of interest; (3) studies in which the primary outcome was the risk of COVID-19 infection, severity, and mortality (given the number of cases as a cross-tabulated table). Studies were excluded if any of the following criteria were met: (1) non-human studies; (2) non-observational studies or observational studies without an analytical epidemiologic approach; (3) irrelevant exposure or outcome variables; (4) duplicate or unobtainable abstract/full-text; (5) studies that reported risk estimates (RR, OR, or HR) and their 95% confidence intervals (CIs) without presenting the number of cases.

### Data extraction

MOK and EP excluded reviews, replies, and letters. Research articles with inappropriate or inadequate results for the quantitative analysis were excluded. MOK and EP extracted the data using a standardized data format from studies that gave the number of cases according to vitamin D levels and COVID-19 infection/outcomes as a cross-tabulated table. We did not use the estimates of summary statistics presented in the studies. Any discrepancies were resolved by consensus. For the qualitative analysis, we created an electronic spreadsheet in which the following information was recorded: authors, location, region, study design, sample size, gender, population age, the definition of deficiency and insufficiency of vitamin D, the evaluated outcomes, and whether the study was included in the meta-analysis. Five studies [21-25] in the qualitative analysis were not included in the meta-analysis because they did not contain sufficient information for quantitative analysis.

### Statistical analysis

We used the Mendeley Desktop version 1.19.4 (https://www.mendeley.com/) to remove duplicates and apply the inclusion criteria. Infection with COVID-19, severe COVID-19, and COVID-19 mortality were considered as COVID-19 outcomes in the meta-analyses. Serum levels of vitamin D are classified using definitions of VDD and vitamin D insufficiency according to advisory committees [26]. Vitamin D laboratory measurement units can be converted to each other as follows: 12 ng/mL=30 nmol/L, 20 ng/mL=50 nmol/L, and 30 ng/mL=75 nmol/L. Despite the existence of classifications and definitions of VDD cut-off values in the literature, some of the studies did not utilize these distinctions, as shown in Table 1 [27-47]. In order to create a subgroup according to a common cut-off value, we chose a serum 25(OH)D level of less than 20 ng/mL (50 nmol/L), as suggested in the 2011 Endocrine Society guideline [19], as indicative of deficiency.

We extracted data from included studies that classified the number of cases according to serum vitamin D levels and COVID-19 infection/outcomes to calculate combined OR estimations. We did not use studies that presented vitamin D levels as mean or median values or presented summary statistics such as the OR, RR, HR, and incidence rate ratio (IRR) expressed without information on the number of cases. We created a subgroup of studies that used a cut-off of 25(OH)D< 20 ng/mL (or < 50 nmol/L). We also performed an overall meta-analysis without considering differences in the definition of serum 25(OH)D levels to compare the results.

We examined the heterogeneity and the publication bias of the included studies using the Cochran Q test, the funnel plots, and the Egger test. Heterogeneity was detected in all groups according to the Cochran Q test and the level of heterogeneity was identified using the I^2^ index. Therefore, the Peto random-effect model was used to estimate combined OR values. We generated forest plots to show the detailed representation of all studies based on the OR effect size with 95% CIs. Moreover, since the Egger test detected publication bias in 1 of the meta-analyses conducted in this study, the trim-and-fill adjustment method was performed. All statistical analyses were done using RStudio version 1.2.5019 (https://docs.rstudio.com/) with R for Windows 4.0.3.

### Assesment of methodological quality

The Newcastle-Ottawa Scale (NOS) [48] (Supplementary Material 3) was used to assess the quality and risk of bias of studies. This tool contains 8 customized evaluation sheets with criteria divided into 3 groups: selection, comparability, and outcome. The case representativeness, research methodologies, and study outcomes were all reviewed on the assessment sheet. Different criteria were used to measure quality depending on different research designs. For the assessment of quality, a score of 3 stars or 4 stars in the selection domain and 1 star or 2 stars in the comparability domain and 2 stars or 3 stars in the exposure/outcome domain indicates good quality; a score of 2 stars in the selection domain and 1 star or 2 stars in the comparability domain and 2 stars or 3 stars in the exposure/outcome domain indicates fair quality; a score 0 or 1 star in the selection domain and 0 or 1 star in the comparability domain and 1 star or 2 stars in the exposure/outcome domain indicates poor quality.

### Ethics statement

Additional ethical approval is not required since the meta-analysis studies used metadata from ethically approved research articles.

## RESULTS

**Figure 1.**
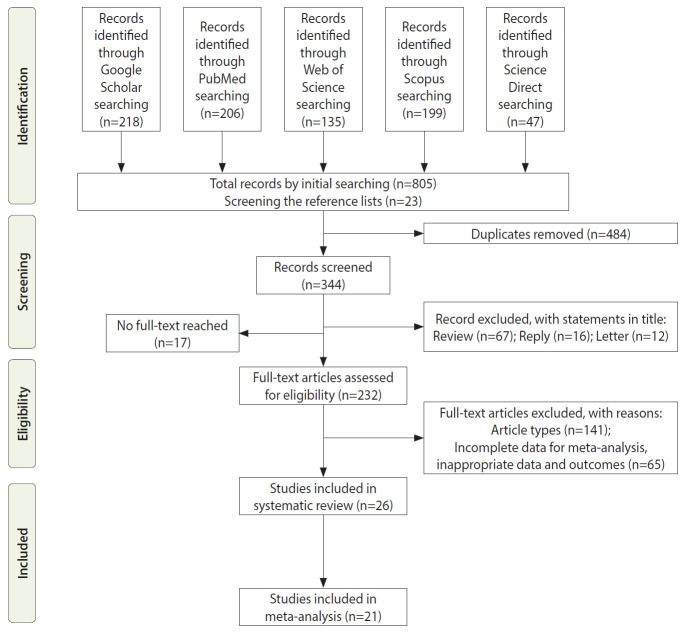
Flowchart of the study selection process.

The initial search yielded 805 articles from the search keywords and 23 articles from screening the reference lists. After the removal of duplicates and exclusion of studies on the basis of their abstracts or through examining their full text, 26 articles were eligible for our systematic review and 21 articles were eligible for the meta-analysis (Figure 1). Five studies were excluded from the meta-analysis because they did not report the data necessary to calculate ORs. Therefore, these studies were only included in the systematic review. The baseline characteristics of the studies are presented in Table 1. The studies were done in Europe (57.7%; 15 studies), Asia (30.8%; 8 studies), and America (11.5%; 3 studies). The total sample size of the 26 studies in the systematic review was 2,277,860. In the 25 studies that reported the gender distribution, the sample size was 1,935,974 (85.0%), of whom 896,444 (46.3%) were men. The summary statistics for the population age were presented in different ways such as mean± standard deviation (SD), and median (minimum-maximun or interquartile range [IQR]) in 15 studies. In 11 studies, the summary statistics were presented separately for each subgroup (case, control, etc.), but not for the overall population. Therefore, we could not obtain the mean or median values of population age from studies, but the light of the information that is available, it is clear that almost the entire population consisted of adults between the ages of 18 years and 85 years. Twelve studies measured 25(OH)D levels using units of ng/mL, 9 studies used nmol/L, 1 study used ng/dL, and 4 studies did not report it. VDD was defined as a 25(OH)D level < 10 ng/mL in 2 studies; < 12 ng/mL in 1 study; < 20 ng/mL in 9 studies; < 25 nmol/L in 3 studies; < 50 nmol/L in 3 studies. One study defined VDD as a 25(OH)D level < 20 ng/dL. In 1 of the studies, the cut-off value of the serum 25(OH)D level was reported as 34.4 nmol/L. This value was expressed as the cohort median, not as deficiency. Vitamin D insufficiency was defined as a 25(OH)D level <20 ng/mL in 1 study, <30 ng/mL in 7 studies, <50 nmol/L in 3 studies, and <75 nmol/L in 1 study.

The COVID-19 outcomes evaluated in the studies were as follows: COVID-19 infection aloe in 8 studies, severity alone in 2 studies, infection and mortality in 2 studies, severity and mortality in 3 studies, infection and severity in 3 studies, infection and hospitalization in 1 study, hospitalization and severity in 1 study, hospitalization and mortality in 1 study. Among the studies that examined 3 outcomes, 3 studies reported COVID-19 infection, severity, and mortality; 1 study reported COVID-19 infection, hospitalization, and severity; and 1 study reported COVID-19 hospitalization, severity, and mortality. Due to the limitations of the studies, we could not extract data on all the evaluated outcomes. Therefore, in some cases, the examined outcomes in meta-analyses and appropriate samples for the meta-analyses were less than the corresponding numbers in the original studies (Table 2).

The NOS was used to assess the quality of the studies included in the meta-analyses [48] (Supplementary Material 3).

The primary hypothesis was that there is a relationship between low vitamin D levels, defined as 25(OH)D < 20 ng/mL or 50 nmol/L, and COVID-19 infection/outcomes. However, since the units of laboratory measurement and levels of measurement were different across the studies included in the meta-analyses, we also applied an overall meta-analysis that included all studies to show the degree to which these differences affected the findings. The results obtained from the overall meta-analyses are presented in the Supplementary Materials 4-7. We performed 6 meta-analyses, as follows:

Vitamin D and COVID-19 infection meta-analysis (D-CIMA) for 25(OH)D < 20 ng/mL or 50 nmol/L, with 8 included studies; vitamin D and COVID-19 infection meta-analysis (D-CIMA_Overall_) for all measurement units, with 11 included studies; vitamin D and COV ID-19 mortality meta-analysis (D-CSMA) for 25(OH)D <20 ng/mL or 50 nmol/L, with 9 included studies; vitamin D and COVID-19 severity meta-analysis (D-CSMAOverall) for all measurement units, with 13 included studies); vitamin D and COVID-19 mortality meta-analysis (D-CMMA) for 25(OH)D <20 ng/mL or 50 nmol/L, with 5 included studies; and vitamin D and COVID-19 mortality meta-analysis (D-CMMAOverall) for all measurement units, with 8 included studies.

The sample sizes were: 202,561 and 203,962 in D-CIMA and D-CIMA_Overall_, 2,120 and 3,564 in D-CSMA and D-CSMAOverall, and 617 and 996 in D-CMMA and D-CMMAOverall, respectively. Since multiple outcomes were examined in the same sample in some studies included in the meta-analyses, there were overlapping samples. Considering these samples, the total sample size was 205,869. For the distribution of samples (Supplementary Material 8).

In 1 study included in D-CIMA [33], we could not find the distribution of gender. Therefore, the number of samples for which the gender distribution could be determined was 19,615, of whom 8,750 (44.6%) were men. As mentioned before, the summary statistics for age were presented in different ways such as mean±SD and median (minimum-maximum or IQR). Therefore, we could not provide summary statistics about the age of the population from the studies, but in the light of available information, we can say that almost the entire population consisted of adults (18-85 years).

**Figure 2.**
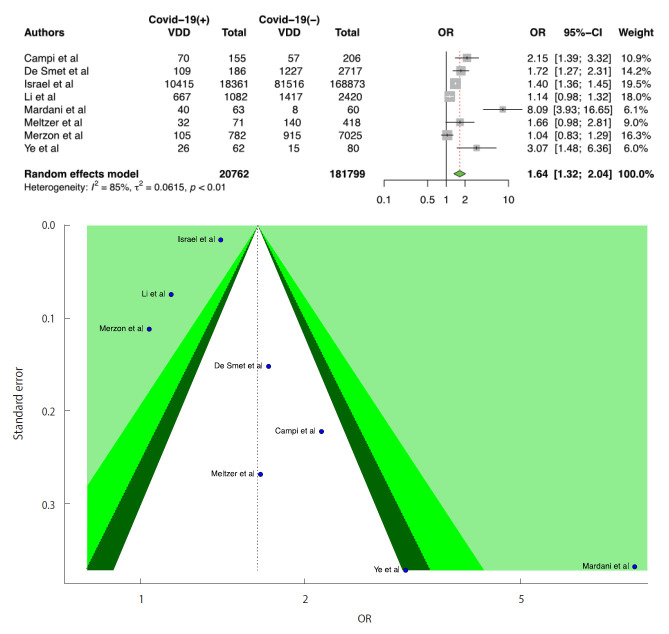
Forest plot of the random-effect meta-analysis and contour-enhanced funnel plot to assess causes of funnel plot asymmetry for vitamin D and coronavirus disease 2019 (COVID-19) infection in the meta-analysis for serum 25(OH)D levels <20 ng/mL or 50 nmol/L. For the CI, the light-green area indicates p<0.01, the green area indicates 0.01≤p<0.05, and the dark-green area indicates 0.05≤p<0.1. The Egger test p-value was 0.399. VDD, vitamin D deficiency; OR, odds ratio; CI, confidence interval.

We included 8 studies in the D-CIMA meta-analysis. All 8 studies reported that there was a significant positive relationship between VDD and the risk of infection. We also found that there was a significant positive relationship between COVID-19 infection and VDD (OR, 1.64; 95% CI, 1.32 to 2.04; p<0.001). There was no publication bias in the 8 studies according to the Egger test (p=0.399). For heterogeneity, the following values were obtained: I^2^=85.4%; 95% CI, 73.2 to 92.1 and τ^2^=0.06; 95% CI, 0.05 to 1.02 (Cochran Q, p<0.001). We generated forest and funnel plots (Figure 2).

We included 11 studies in the D-CIMA_Overall_ meta-analysis. All studies except for 1 reported that there was a significant positive relationship between VDD and COVID-19 infection. According to D-CIMA_Overall_ results, a low serum level of vitamin D was positively associated with COVID-19 infection (OR, 1.86; 95% CI, 1.51 to 2.30; p<0.001). There was no publication bias in the 11 studies according to the Egger test (p=0.091). For heterogeneity, the following values were obtained: I^2^=87.0%; 95% CI, 78.7 to 92.1 and τ^2^=0.08; 95% CI, 0.06 to 0.76 (Cochran Q, p<0.001). We presented forest and funnel plots (Supplementary Material 4). Considering the D-CIMA and D-CIMA_Overall_ results, we should note that the combined OR values are different. This is a remarkable finding that demonstrates the importance of distinction according to serum vitamin D level.

**Figure 3.**
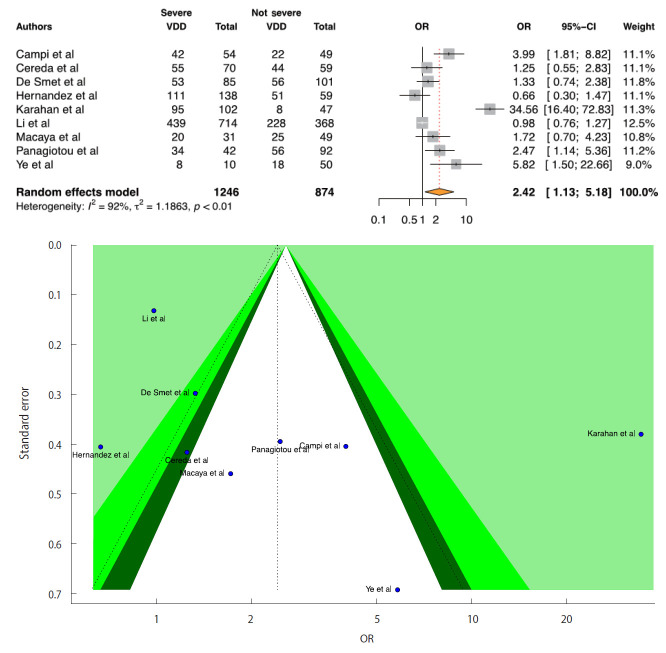
Forest plot of the random-effect meta-analysis and contour-enhanced funnel plot to assess causes of funnel plot asymmetry for vitamin D and coronavirus disease 2019 (COVID-19) severity in the meta-analysis for serum 25(OH)D levels <20 ng/mL or 50 nmol/L. For the CI, the light-green area indicates p<0.01, the green area indicates 0.01≤p<0.05, and the dark-green area indicates 0.05≤p<0.1. The p-value for the Egger test was 0.054. VDD, vitamin D deficiency; OR, odds ratio; CI, confidence interval.

We included 9 studies in the D-CSMA meta-analysis. All of the studies except for 1 [32] reported that there was a significant positive relationship between VDD and the risk of severe COVID-19. We obtained a significant positive relationship between COVID-19 severity and VDD (OR, 2.42; 95% CI, 1.13 to 5.18; p=0.022). There was no publication bias in the 9 studies according to the Egger test (p=0.064). For heterogeneity, the following values were obtained: I^2^=91.5%; 95% CI, 86.1 to 94.8 and τ^2^=1.18; 95% CI, 0.47 to 5.29 (Cochran Q, p<0.001). We generated forest and funnel plots (Figure 3).

We included 13 studies in the D-CSMAOverall meta-analysis. All studies except for 1 [32] reported that low serum levels of vitamin D were positively associated with COVID-19 severity. There was publication bias in the 13 studies according to the Egger test (p=0.017). Due to the presence of publication bias, we applied the trim-and-fill adjustment method. According to the adjusted results, there was no significant relationship between COVID-19 severity and low serum levels of vitamin D (OR, 1.24; 95% CI, 0.71 to 2.17; p=0.445). This is also a remarkable finding that demonstrates the importance of distinguishing among serum vitamin D levels. The forest and funnel plots for the adjusted method are presented in Supplementary Material 5. For heterogeneity in the adjusted method, the following values were obtained: I^2^=92.8%; 95% CI, 90.2 to 94.7 and τ^2^=1.37; 95% CI, 0.88 to 3.98 (Cochran Q, p<0.001). Moreover, we also provided the overall results without applying the trim-and-fill adjustment to show that there is a difference in the results (Supplementary Material 6).

Considering the D-CSMA and D-CSMAOverall results, we should note that conducting an analysis with all data leads to misleading results, such as finding that there is no relationship when one actually exists. This is also a remarkable finding that demonstrates the importance of distinguishing among serum vitamin D levels.

**Figure 4.**
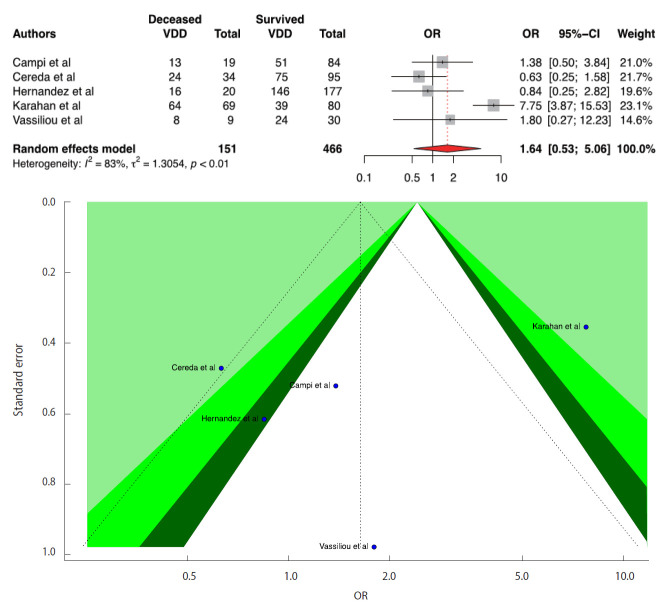
Forest plot of the random-effect meta-analysis and contour-enhanced funnel plot to assess causes of funnel plot asymmetry for vitamin D and coronavirus disease 2019 (COVID-19) mortality in the meta-analysis for serum 25(OH)D levels <20 ng/mL or 50 nmol/L. For the CI, the light-green area indicates p<0.01, the green area indicates 0.01≤p<0.05, and the dark-green area indicates 0.05≤p<0.1. The p-value for the Egger test was 0.528. VDD, vitamin D deficiency; OR, odds ratio; CI, confidence interval.

We included 5 studies in the D-CMMA meta-analysis. Three studies reported a significant positive relationship between VDD and COVID-19 mortality. In contrast to the results reported in 3 studies, we found that there was no significant relationship between COVID-19 mortality and VDD (OR,1.64; 95% CI, 0.53 to 5.06; p=0.390). There was no publication bias in the 5 studies according to the Egger test (p=0.911). For heterogeneity, the following values were obtained: I^2^=82.6%; 95% CI, 60.0 to 92.4 and τ^2^=1.30; 95% CI, 0.12 to 10.51 (Cochran Q, p<0.001). Forest and funnel plots are presented in Figure 4.

We included 8 studies in the D-CMMAOverall meta-analysis. While 4 studies of the 8 studies reported that low serum levels of vitamin D were positively associated with COVID-19 mortality, the rest of the studies reported that there was no significant relationship. We found that low serum levels of vitamin D were not associated with COVID-19 mortality (OR, 1.58; 95% CI, 0.76 to 3.27; p=0.211). There was no publication bias in the 8 studies according to the Egger test (p=0.909), and the following values were found for heterogeneity: I^2^=70.8%; 95% CI, 39.7 to 85.9 and τ^2^=0.71; 95% CI, 0.00 to 3.02 (Cochran Q, p=0.001). The forest and funnel plots are presented in Supplementary Material 7. All the results obtained from the meta-analyses are presented in Table 3.

## DISCUSSION

Although vaccination has started in many countries with vaccines that have been approved for emergency use, it seems that COVID-19 will continue to threaten public health for a long time. Therefore, there is a need to protect individuals from COVID-19, which is reported to have become more contagious due to mutations [49] and to reduce the risk of severe disease, consequently reducing the mortality rate. In addition to vaccination as a preventive measure against COVID-19, it has also been recommended to use supplements that strengthen the immune system [50,51]. From this point of view, the purpose of this study was to determine the possible effect of VDD on COVID-19 infection and outcomes due to its antiviral properties and to provide additional evidence in the literature. For this purpose, we conducted a systematic review of 26 studies and 3 meta-analyses of 21 studies, with careful attention paid to the laboratory measurement units for vitamin D and the measured serum 25(OH)D levels. We should emphasize that it is important to construct hypotheses based on a clear definition of VDD with serum 25(OH)D levels. However, studies have defined VDD using different serum 25(OH)D levels; for instance, a serum 25(OH)D level of 23 ng/mL would be defined as VDD in one study but as vitamin D insufficiency in another study. Therefore, analyzing findings in relation to VDD as defined in studies without accounting for how VDD was defined in terms of serum 25(OH)D levels may lead to misleading results. Our results have shown how important it is to make this distinction. Therefore, to the best of our knowledge, this meta-analysis study is the most comprehensive study to date in terms of the number of included studies, inclusion criteria, sample size, and well-defined hypotheses. However, our study has some limitations. In our study design, we did not include OR values that were presented in logistic regression models in the included studies to show the pure effect of VDD on the examined outcomes. Since the logistic models in the studies were established with different explanatory variables, the presented OR values cannot represent the same effect. We would have liked to provide evidence for comorbidities, treatment, and hospitalization through meta-analyses, but it was not possible to extract data suitable for our study design. The difficulties arising from the designs of the studies included in the meta-analyses during data extraction are presented in Table 3.

According to the results from the D-CIMA meta-analysis, people with serum 25(OH)D levels below 20 ng/mL or 50 nmol/L are 1.64 times more likely to be infected with COVID-19. COVID-19 infection then D-CSMA meta-analysis, we found that people with a serum 25(OH)D level below 20 ng/mL or 50 nmol/L were 2.42 times more likely to have severe COVID-19. However, the DCMMA meta-analysis showed that low vitamin D levels had no effect on COVID-19 mortality. It is also important to discuss the results of the overall meta-analyses presented in the Supplementary Materials 4-7 along with our main findings. When 11 studies were combined without a distinction based on serum 25(OH)D levels in D-CIMA_Overall_, an OR of 1.86 was obtained (Supplementary Material 4). This is a misleading result that can be interpreted as showing that VDD will increase the risk of infection from COVID-19 to a greater extent. Similarly, when 13 studies were combined without discrimination according to serum 25(OH)D levels in D-CSMAOverall, no significant relationship was found between VDD and the risk of having severe COVID-19 (Supplementary Material 5). This result would cause the existing relationship to be ignored and the effect of vitamin D on preventing severity to be neglected. Although we could not find a significant relationship between VDD and mortality, we think that similar results also would be obtained for D-CMMA. However, due to the fact that the available data were limited, we could not provide sufficient evidence for the effect of vitamin D on mortality (Figure 4) (Supplementary Material 7).

In our opinion, existing meta-analyses on the subject cannot provide reliable and sufficient evidence. Pereira et al. [16] performed a meta-analysis including 21 studies with 8,176 samples to examine the relationships of VDD with COVID-19 infection, severity, hospitalization, and mortality. They found that the relationship between VDD and COVID-19 infection is not significant (OR, 1.21; 95% CI, 0.83 to 1.60), but significant relationships between VDD and severity (OR, 1.65; 95% CI, 1.30 to 2.09), hospitalization (OR, 1.81; 95% CI, 1.42 to 2.21), and mortality (OR, 1.82; 95% CI, 1.06 to 2.58). However, we identified some problems such as incorrect referencing, inconsistencies in the number of included studies throughout the text, the use of OR values in the meta-analyses that were not in the relevant studies, and incorrect presentation of the characteristics of the included studies. As an example, they included in the same meta-analysis Hastie et al. [23]’s article and corrigendum [52] of Hastie et al. [53]. Moreover, it is unclear how they obtained OR values from the corrigendum, where only a correction table about population characteristics was presented. In addition, although Hastie et al. [23] presented an IRR value for VDD < 25 nmol/L, Pereira et al. [16] used it as an OR value for VDD < 50 nmol/L. For more detail, we refer the reader to Pamukçu & Kaya [54]’s letter to the editor. Considering the other included studies, Meltzer et al. [41] and Darling et al. [22] presented their findings using RRs and ORs, respectively. It seems that Pereira et al. [16] combined different summary statistics such as IRRs, ORs, and RRs. Therefore, the findings obtained from that study are doubtful. Munshi et al. [17] conducted a meta-analysis study combining only 6 studies with a relatively small sample (n=376). They reported that patients with a poor prognosis had significantly lower serum levels of vitamin D than those with a good prognosis, with an adjusted standardized mean difference of -0.58 (95% Cl, -0.83 to -0.34; p<0.001). In addition, they presented a subgroup meta-analysis in which they examined differences in vitamin D according to regions. Here, it was determined that a subgroup was inappropriately formed from a single study. Chen et al. [18] conducted a meta-analysis including 6 studies with 377,265 samples to examine the relationships between VDD and COVID-19 infection, hospitalization, and mortality. They found a significant association for COVID-19 infection (OR, 1.47; 95% CI, 1.09 to 1.97) and hospitalization (OR, 1.83; 95% CI, 1.22 to 2.74), while they did not find a meaningful relationship for mortality (OR, 2.73; 95% CI, 0.27 to 27.61). Although subgroup analyses were performed according to serum 25(OH)D levels < 20 ng/mL and < 30 ng/mL, the results were given in the overall estimate. The problem of the subgroup analysis being performed with a single study was also identified here. When we consider our findings together with the results obtained from these studies, we can say that we put forth more credible evidence regarding the relationship between VDD and COVID-19 infection and severity.

As we present in the Results section, our findings support previous results in the literature, according to which vitamin D supplementation has a protective effect against acute respiratory infections [55]. It has been reported that antigen-presenting cells such as macrophages and dendritic cells play a role in the synthesis of the active form of vitamin D and that macrophages and dendritic cells can be affected by vitamin D. Studies have reported that active vitamin D (1,25[OH]_2_D) synthesis is reduced during VDD, so the immune response, including innate immune function, will be impaired. In addition, vitamin D shows antimicrobial and antiviral activity by increasing the expression of cathelicidin/defensin. Cathelicidin and defensin contributes to host defense by stimulating the expression of antiviral cytokines and chemokines involved in the recruitment of monocytes/macrophages, natural killer cells, neutrophils, and T cells. Cellular production of cathelcidin and defensin depends on the vitamin D receptor and *CYP27B1*, the expression of which is enhanced following interactions of pathogens with membrane pattern recognition receptors, such as toll-like receptor and toll-like receptor 2. The above-mentioned mechanism explains the role of vitamin D in combating respiratory viruses [56]. In the early stage of infection, it limits viral entry and replication by increasing cathelicidin and defensin expression in the respiratory epithelium [57]. In COVID-19, pneumonia and ARDS have been identified as responsible for severe disease. Many studies have reported the protective effects of vitamin D against pneumonia, cytokine hyperproduction, and many conditions associated with ARDS [58,59]. It has also been reported that VDD is directly associated with the risk of acute respiratory failure [60,61]. In a rat study, it was reported that vitamin D supplementation reduced lung damage and attenuated disease severity in rats with ARDS [62]. Although sufficient data have not been found for COVID-19, vitamin D has recently been recommended as a drug to treat lung damage in pneumonia caused by influenza A virus [63]. In light of the evidence we have obtained, vitamin D supplementation can be recommended to reduce the severity of COVID-19 disease.

The fact that COVID-19 mortality rates differ between countries and that mortality rates are lower in the Southern Hemisphere has attracted attention to the relationship between VDD and death. In a study conducted in European countries, which are located in the Northern Hemisphere and do not have sufficient sunlight in winter, it has been reported that average vitamin D levels are associated with mortality, especially in countries with a high prevalence of VDD such as Italy and Spain where the mortality due to COVID-19 has been high [64]. Although it has been found that benefiting from sunlight reduces influenza infection and related mortality, no studies in the literature have elucidated the effect of vitamin D supplementation and seasonal changes on mortality associated with COVID-19 [51,65]. The cytokine storm, which causes hyperinflammation and tissue damage, has been found to be responsible for the mortality associated with COVID-19 [66]. In the literature, it has been emphasized that vitamin D can be an important agent in preventing cytokine storm and ARDS. Based on this information, it is reasonable to hypothesize that vitamin D can reduce the mortality rate due to COVID-19. However, the current literature could not provide adequate data to support this hypothesis in our meta-analysis. We should note that large-scale and multi-center randomized controlled studies are still needed to determine the effectiveness of vitamin D as a treatment to reduce disease severity and mortality.

### CONCLUSION

Despite the fact that the vaccination has started in many countries, it appears that COVID-19 will continue to threaten public health for a long time. Hence, in addition to the vaccine, the usage of immune-supporting supplements may be beneficial. The main purpose of this study was to synthesize evidence on the possible effect of low serum vitamin D levels (25[OH]D < 20 ng/mL or 50 nmol/L) on COVID-19 infection and outcomes. According to our results, VDD may increase the risk of COVID-19 infection and the potential for severe disease. Therefore, vitamin D supplementation may be added to prevention and treatment protocols for COVID-19. It should be noted that current measures to reduce transmission, such as frequent hand washing, wearing a mask, physical separation, air circulation, and avoiding crowded locations or enclosed spaces, continue to work against new varieties by limiting viral transmission and thereby reducing the virus’s ability to mutate. Vaccines are a vital tool in the fight against COVID-19, and employing extant tools will have significant public health and lifesaving benefits.

## Figures and Tables

**Table 1.** The basic characteristics of the studies included in the systematic review and meta-analyses

Study	Location	Region	Study design	Sample size	Gender (men), n (%)	Population age mean±SD or median [Min-Max or IQR]	The definition of VDD and/or VDIS	Outcomes evaluated in the study	Included in the meta-analysis
Abdollahi et al. [27]	Iran	Asia	Case-control	402	132 (32.8)	47.1±15.3	VDD<10 ng/mL	I	Yes
							VDIS<30 ng/mL		
Baktash et al. [28]	UK	Europe	Cross-sectional	105	57 (54.2)	81 [65-102]	VDD<30 nmol/L	I-M	Yes
Campi et al. [29]	Italy	Europe	Cohort	361	243 (67.0)	66 [54–78]	VDD<50 nmol/L	I-S-M	Yes
Cereda et al. [30]	Italy	Europe	Cohort	129	70 (54.3)	77 [65–85]	VDD<20 ng/mL	S-M	Yes
							VDIS<30 ng/mL		
D’Avolio et al. [21]	Switzerland	Europe	Case-control	1,484	682 (45.9)	NR	NR	I	No
Darling et al. [22]	UK	Europe	Case-control	1,303	713 (54.7)	57.7±8.7	NR	I	No
De Smet et al. [31]	Belgium	Europe	Cross-sectional	2,903	1,108 (38.1)	NR	VDD<20 ng/mL	I-S	Yes
Hastie et al. [23]	UK	Europe	Cohort	341,484	NR	NR	VDD<25 nmol/L	I-M	No
							VDIS<50 nmol/L		
Hernández et al. [32]	Spain	Europe	Case-control	413	253 (61.2)	NR	VDD<20 ng/mL	I-S-M	Yes
Im et al. [24]	Korea	Asia	Case-control	200	84 (42.0)	52.3±20.3	VDD<20 ng/dL	I-S	No
Israel et al. [33]	Israel	Asia	Case-control	576,455	271,601 (47.1)	NR	VDD<50 nmol/L	I	Yes
Karahan and KatKat [34]	Turkey	Europe	Cross-sectional	149	81 (54.3)	63.5±15.3	VDD<20 ng/mL	S-M	Yes
							VDIS<30 ng/mL		
Katz et al. [25]	USA	America	Case-control	987,849	455,458 (46.1)	NR	NR	I	No
Li et al. [35]	UK	Europe	Case-control	353,299	161,298 (45.6)	67.7±8.1	VDD<25 nmol/L	I-H-S	Yes
							VDIS<50 nmol/L		
Livingston et al. [36]	UK	Europe	Cohort	104	39 (37.5)	68.5±18.3	VDSL<34.4 nmol/L	I	Yes
Luo et al. [37]	China	Asia	Cross-sectional	895	405 (45.2)	NR	VDD<30 nmol/L	I-S-M	Yes
Macaya et al. [38]	Spain	Europe	Cohort	80	35 (43.7)	NR	VDD<20 ng/mL	S	Yes
Maghbooli et al. [39]	Iran	Asia	Cross-sectional	235	144 (61.3)	58.7±15.2	VDD<20 ng/mL	H-S-M	Yes
							VDIS<30 ng/mL		
Mardani et al. [40]	Iran	Asia	Cross-sectional	123	65 (52.8)	42.1±14,9	VDD<10 ng/mL	I	Yes
							VDIS<30 ng/mL		
Meltzer et al. [41]	USA	America	Case-control	489	123 (25.0)	49.2±18.4	VDD<20 ng/mL	I	Yes
Mendy et al. [42]	USA	America	Cohort	689	365 (53.0)	49.5 [35.2–67.5]	NR	H-S	Yes
Merzon et al. [43]	Israel	Asia	Case-control	7,807	3,234 (41.4)	41.4±NR	VDD<20 ng/mL	I-H	Yes
							VDIS<30 ng/mL		
Panagiotou et al. [44]	UK	Europe	Cohort	134	73 (54.4)	68.7±14.0	VDD<25 nmol/L	S	Yes
							VDIS<50 nmol/L		
Radujkovic et al. [45]	Germany	Europe	Cohort	185	95 (51.0)	60 [49–70]	VDD<12 ng/mL	S-M	Yes
							VDIS<20 ng/mL		
Vasiliou et al. [46]	Greece	Europe	Cohort	39	31 (79.4)	61.5±13.2	VDD<20 ng/mL	H-M	Yes
							VDIS<30 ng/mL		
Ye et al. [47]	China	Asia	Case-control	142	55 (38.7)	NR [0.1-85]	VDD<50 nmol/L	I-S	Yes
							VDIS<75 nmol/L		

SD, standard deviation; Min, minimun; Max, maximun; IQR, interquartile range; NR, not reported; VDD, vitamin D deficiency; VDIS, vitamin D insufficiency; VDSL, vitamin D serum level; H, hospitalization; I, COVID-19 infection; S, severity; M, mortality.

**Table 2.** Limitations and additional characteristics of the included studies

Study	Sample size in the study	Sample size included in meta-analyses			Outcomes evaluated in the study	Outcomes evaluated in the meta-analyses	Findings in the study^1^	Limitations in extracting data
		D-CIMA	D-CSMA	D-CMMA				
Abdollahi et al. [27]	402	402	–	–	I	I	I(+)	VDD was defined as <10 ng/mL, but the sample distribution for VDD was not suitable for statistical analysis; A statistical comparison was made for VDIS <30 ng/mL; Therefore, the data belonging to VDIS were included in the meta-analysis, and the measurement unit was assigned as “other”
Baktash et al. [28]	105	–	–	70	I-M	M	I(+); M(-)	There was uncertainty in the definition of comparison groups for COVID-19 infection; We sent an email to the corresponding author, but we did not receive a reply; Therefore, COVID-19 infection data were not included in the D-CIMA
Campi et al. [29]	361	361	103	103	I-S-M	I-S-M	I(+); S(+); M(+)	There was no limitation in data extraction
Cereda et al. [30]	129	–	129	129	S-M	S-M	S(+); M(+)	The clinical outcomes were severe pneumonia, admission to the ICU, and in-hospital mortality; The ICU admission data were not appropriate for statistical analysis (0 observed cases); Therefore, we extracted data from severe pneumonia for the analysis of severity
D’Avolio et al. [21]	1,484	–	–	–	I	–	I(+)	The definition of VDD was not available; There was no adequate information dealing with descriptive statistics; VDD was presented as median (IQR) and the findings were presented graphically; Since we extracted the data based on the number of cases, we did not include it in the meta-analysis In the study, the date of the data received from UK Biobank for the COVID-19 (-) control group was long ago; There was no VDD definition and they used quartile values of VDD; Since we extracted the data based on the number of cases, we did not include it in the meta-analysis
Darling et al. [22]	1,303	–	–	–	I	–	I(-)	In the study, the date of the data received from UK Biobank for the COVID-19 (-) control group was long ago; There was no VDD definition and they used quartile values of VDD; Since we extracted the data based on the number of cases, we did not include it in the meta-analysis
De Smet et al. [31]	2,903	2,903	186	–	I-S	I-S	I(+); S(+)	In the study, chest CT was performed for all COVID-19 patients to determine the disease stage; They classified the patients as stage 1 (early stage), stage 2 (progressive stage), and stage 3 (peak stage); We chose severe cases from stage 3 and non-severe cases from stage 1-2
Hastie et al. [23]	341,484	–	–	–	I-M	–	I(-); M(-)	There was no adequate information dealing with descriptive statistics; The findings were presented by using HR and IRR values; Since we extracted the data based on the number of cases, we did not include it in the meta-analysis
Hernández et al. [32]	413	–	197	197	I-S-M	S-M	I(+); S(-); M(-)	For COVID-19 infection, VDD was presented as mean (±SD); Since the number of cases was not reported, we could not include it in D-CIMA
Im et al. [24]	200	–	–	–	I-S	–	I(+); S(+)	In the study, the unit of ng/dL was used for laboratory measurements of serum 25(OH)D levels, and VDD was defined as 25(OH)D<20 ng/dL; We converted 20 ng/dL to 0.2 ng/mL; Since we thought there was an inconsistency, we could not include these data in the meta analyses
Israel et al. [33]	576,455	187,234	–	–	I	I	I(+)	Age matching was performed between the case and control groups, but single summary statistics for the population were not presented
Karahan and Katkat [34]	149	–	149	149	S-M	S-M	S(+); M(+)	There was no limitation in data extraction
Katz et al. [25]	987,849	–	–	–	I	–	I(+)	There was no adequate information dealing with descriptive statistics; The findings were presented as ORs; Since we extracted the data based on the number of cases, we did not include it in the meta-analysis
Li et al. [35]	353,299	3,502	1,082	–	I-H-S	I-S	I(+); H(+); S(+)	This study defined hospitalization as one record of origin (whether the patient s tested positive or not); In the study, hospitalized, confirmed, and severe COVID-19 cases were compared with community controls; We think that this study design is inappropriate; For this reason, we extracted data on infections from patients who presented to the hospital with suspected COVID-19 and confirmed COVID-19, and for the severity from patients with confirmed and severe COVID-19
Livingston et al. [36]	104	104	–	–	I	I	I(-)	The definition of VDD was not available; Statistical comparisons were performed for 25(OH)D <34.4 nmol/L; Therefore, the data belonging to this distinction were included in the meta-analysis, and the measurement unit was assigned as “other”
Luo et al. [37]	895	895	335	74	I-S-M	I-S-M	I(+); S(+); M(-)	There was no limitation in data extraction
Macaya et al. [38]	80	–	80		S	S	S(+)	There was no limitation in data extraction
Maghbooli et al. [39]	235	–	235	235	H-S-M	S-M	H(+); S(+); M(+)	There was no limitation in data extraction; Although the authors defined VDD as 25(OH)D<20 ng/mL, the dataset that could be extracted from the article belonged to the category of 25(OH)D<30 ng/mL; Therefore, it was included in the overall analysis
Mardani et al. [40]	123	123	–	–	I	I	I(+)	We extracted data from their supplementary file according to 25(OH)D<20 ng/mL
Meltzer et al. [41]	489	489	–	–	I	I	I(+)	There was no limitation in data extraction
Mendy et al. [42]	689	–	689	–	H-S	S	H(+); S(+)	The definition of VDD was not available; Therefore, the measurement unit was assigned as “other” in the D-CSMA
Merzon et al. [43]	7,807	7,807	–	–	I-H	I	I(+); H(+)	There was no limitation in data extraction
Panagiotou et al. [44]	134	–	134	–	S	S	S(+)	The authors classified the patients as admitted to the ITU and non-ITU wards;
								When we extracted data, we chose patients admitted to non-ITU wards as non-severe and those admitted to ITU wards as severe; A statistical comparison was made for VDIS <50 nmol/L; Therefore, the data belong to VDIS were included in the D-CSMA
Radujkovic et al. [45]	185	–	185	–	S-M	S	S(+); M(+)	The authors classified patients as inpatients and outpatients; When we extracted data, we chose the outpatients as non-severe and the inpatients as severe according to their definition
Vassiliou et al. [46]	39	–	–	39	H-M	M	H(-); M(-)	There was no limitation in data extraction
Ye et al. [47]	142	142	60	–	I-S	I-S	I(+); S(+)	There was no limitation in data extraction
Total	2,277,860	203,962	3,564	996				

COVID-19, coronavirus disease 2019; D-CIMA, vitamin D and COVID-19 infection meta-analysis; D-CSMA, vitamin D and COVID-19 severity meta-analysis; D-CMMA, vitamin D and COVID-19 mortality meta-analysis; I, COVID-19 infection; S, severity; M, mortality; H, hospitalization; VDD, vitamin D deficiency; ICU, intensive care unit; IQR, interquartile range; CT, computed tomography; HR, hazard ratio; IRR, incidence rate ratio; SD, standard deviation; OR, odds ratio; ITU, intensive therapy unit; VDIS, vitamin D insufficiency.

1 (+), indicates that the association of low levels of vitamin D and I, S, M and H was statistically significant; (-) indicates that the association of low levels of vitamin D and I, S, M and H was not statistically significant.

**Table 3.** P-values of tests of publication bias, heterogeneity, and meta-analysis findings and bias scores for the Egger test

Meta-analyses	Publication bias	Heterogeneity	Quantifying heterogeneity	Findings of meta-analyses	
	Egger test (p-value)	Cochran Q test (p-value)	I^2^ (95% CI), %/τ^2^ (95% CI)	Peto random-effect model	
				OR (95% CI)	p-value
D-CIMA for serum 25(OH)D levels <20 ng/mL and 50 nmol/L	0.399	<0.001	85.4 (73.2, 92.1)/0.06 (0.05, 1.02)	1.64 (1.32, 2.04)	<0.001
D-CIMA_Overall_ for serum 25(OH)D levels with all different measurement units	0.091	<0.001	87.0 (78.7, 92.1)/0.08 (0.06, 0.76)	1.86 (1.51, 2.30)	<0.001
D-CSMA for serum 25(OH)D levels <20 ng/mL and 50 nmol/L	0.064	<0.001	91.5 (86.1, 94.8)/1.18 (0.47. 5.29)	2.42 (1.13, 5.17)	0.022
D-CSMA_Overall_ for serum 25(OH)D levels with all different measurement units	0.017	<0.001	92.8 (90.2, 94.7)^1^/1.37 (0.88, 3.98)^1^	1.24 (0.71, 2.17)1	0.445
D-CMMA for serum 25(OH)D levels <20 ng/mL and 50 nmol/L	0.911	<0.001	82.6 (60.1, 92.4)/1.30 (0.12, 10.51)	1.64 (0.53, 5.06)	0.390
D-CMMA_Overall_ for serum 25(OH)D levels with all different measurement units	0.909	0.001	70.8 (39.7, 85.9)/0.71 (0.00, 3.02)	1.58 (0.76, 3.27)	0.211

D-CIMA, vitamin D and COVID-19 infection meta-analysis; D-CSMA, vitamin D and COVID-19 severity meta-analysis; D-CMMA, vitamin D and COVID-19 mortality meta-analysis; COVID-19, coronavirus disease 2019; OR, odds ratio; CI, confidence interval.

1 Trim-and-fill method applied.

## SUPPLEMENTARY MATERIALS

Korean version is available at https://www.e-epih.org/.
