# Supplementary material for: The role of vitamin D deficiency on COVID-19: a systematic review and meta-analysis of observational studies
Source: Epidemiol Health. 2021 Sep 23;43:e2021074. doi: 10.4178/epih.e2021074 (PMC8769802; doi:10.4178/epih.e2021074)
Supplement: Supplementary file 2 [file epih-43-e2021074-suppl2.docx]

Supplementary Material 2. Database search strategies and results between 01.01.2020 and 15.12.2020

| Database | Search Strategy | Items Found |
| --- | --- | --- |
| Google Scholar | allintitle: and "Covid 19" "vitamin D"  allintitle: and "SARS-CoV-2" "vitamin D"  allintitle: and "Coronavirus Disease" "vitamin D" | 218 |
| PubMed | ("vitamin D"[Title] AND "covid-19"[Title]) AND (("2020/01/01"[Date - Publication] : "2020/12/15"[Date - Publication]))  ("vitamin-D"[Title] AND "SARS-CoV-2"[Title]) AND (("2020/01/01"[Date - Publication] : "2020/12/15"[Date - Publication]))  ("vitamin D"[Title] AND "Coronavirus Disease"[Title]) AND (("2020/01/01"[Date - Publication] : "2020/12/15"[Date - Publication])) | 206 |
| Science Direct | Year: 2020, Title: "Vitamin D" and "Covid-19"  Year: 2020, Title: "Vitamin D" and " SARS-CoV-2"  Year: 2020, Title: "Vitamin D" and " Coronavirus Disease " | 47 |
| Scopus | **TITLE** **(** ***"vitamin D"*** **AND** ***"Covid-19"*** **)** **AND** **PUBYEAR** **=** ***2020***  **TITLE ( *"vitamin D"*  AND  *"SARS-CoV-2"* )  AND  PUBYEAR  =  *2020***  **TITLE ( *"vitamin D"*  AND  *"*** ***Coronavirus Disease "* )  AND  PUBYEAR  =  *2020*** | 199 |
| Web of Science | TI=(Vitamin D AND Coronavirus Disease) OR TI=(Vitamin D AND SARS-CoV-2) OR TI=(Vitamin D AND Covid-19)  *Indexes=SCI-EXPANDED, SSCI, A&HCI, CPCI-S, CPCI-SSH, BKCI-S, BKCI-SSH, ESCI Timespan=2020* | 135 |
