# Supplementary material for: The role of vitamin D deficiency on COVID-19: a systematic review and meta-analysis of observational studies
Source: Epidemiol Health. 2021 Sep 23;43:e2021074. doi: 10.4178/epih.e2021074 (PMC8769802; doi:10.4178/epih.e2021074)
Supplement: Supplementary file 3 [file epih-43-e2021074-suppl3.docx]

Supplementary Material 3. Newcastle-Ottawa quality assessment scale for included studies

|  | Selection | | | | Comparability | | Exposure/Outcome | | | Quality |
| --- | --- | --- | --- | --- | --- | --- | --- | --- | --- | --- |
| Case Control Studies | Adequate case definition | Representativeness of cases | Selection of controls | Definition of controls | Comparability of cases and controls on the basis of design or analysis | | Assessment of exposure | Same method of ascertainment for cases and controls | Non-response rate |  |
| Abdollahi et. al. [27] | * | - | * | * | - | - | * | * | - | Poor |
| Hernandez et. al. [33] | * | * | * | - | * | * | * | * | * | Good |
| Israel et. al. [34] | * | * | * | - | * | * | * | * | * | Good |
| Li et. al. [36] | * | * | * | - | - | - | * | * | * | Poor |
| Meltzer et. al. [42] | * | - | * | * | * | * | * | * | - | Good |
| Merzon et. al. [44] | * | * | * | * | * | * | * | * | - | Good |
| Ye et. al. [49] | * | * | * | * | - | - | * | * | - | Poor |

|  | Selection | | | | Comparability | | Exposure/Outcome | | Quality |
| --- | --- | --- | --- | --- | --- | --- | --- | --- | --- |
| Cross-Sectional Studies | Representativeness of the sample | Sample size | Non-respondents | Assessment of exposure | Comparability of different outcome groups on the basis of design or analysis | | Assessment of outcome | Statistical test |  |
| Baktash et. al. [29] | - | - | - | - | * | * | ** | * | Poor |
| De Smet et. al. [32] | - | - | - | * | * | - | ** | * | Poor |
| Karahan et. al. | - | - | - | * | - | - | ** | * | Poor |
| Luo et. al. [38] | - | - | - | * | * | * | ** | * | Poor |
| Maghbooli et. al. [40] | - | - | - | * | - | - | ** | * | Poor |
| Mardani et. al. [41] | - | - | - | * | - | - | ** | - | Poor |

|  | Selection | | | | Comparability | | Exposure/Outcome | | | Quality |
| --- | --- | --- | --- | --- | --- | --- | --- | --- | --- | --- |
| Cohort studies | Representativeness exposure cohort | Selection of non-exposed cohort | Ascertainment of exposure | Absence of outcome of interested at start of study | Comparability of cohorts on the basis of design or analysis | | Assessment of outcome | Follow-up enough for outcome to occur | Adequacy of follow-up of cohorts |  |
| Alipio et. al. [28] | * | * | * | * | - | - | * | - | * | Poor |
| Campi et. al. [30] | * | - | * | * | * | * | * | * | * | Good |
| Cereda et . al. [31] | - | * | * | * | - | - | * | - | * | Poor |
| Livingston et. al. [37] | * | * | * | * | - | - | * | * | * | Poor |
| Macaya et. al. [39] | - | * | * | - | * | * | * | - | - | Fair |
| Mendy et. al. [43] | * | * | * | * | - | - | * | * | * | Poor |
| Panagiotou et. al. [45] | * | * | * | * | - | - | * | - | * | Poor |
| Radujkovic et. al. [46] | - | * | * | * | - | - | * | * | * | Poor |
| Raharusuna et. al. [47] | * | * | * | * | - | - | * | * | * | Poor |
| Vassiliou et. al. [48] | - | * | * | * | - | - | * | * | * | Poor |
| Assessment of Quality= Good quality: 3 or 4 stars in the selection domain and 1 or 2 stars in the comparability domain and 2 or 3 stars in the exposure/outcome domain. Fair quality: 2 stars in selection domain and 1 or 2 stars in comparability domain and 2 or 3 stars in exposure/outcome domain. Poor quality: 0 or 1 star in selection domain and 0 or 1 star in comparability domain and 1 or 2 stars in exposure/outcome domain. | | | | | | | | | | |
