# Supplementary material for: The role of vitamin D deficiency on COVID-19: a systematic review and meta-analysis of observational studies
Source: Epidemiol Health. 2021 Sep 23;43:e2021074. doi: 10.4178/epih.e2021074 (PMC8769802; doi:10.4178/epih.e2021074)
Supplement: Supplementary file 4 [file epih-43-e2021074-suppl4.docx]

Supplementary Material 4. The forest plot for the D-CIMAOverall

Supplementary Material 4. The funnel plot for the studies included in D-CIMAOverall
