# Supplementary material for: The role of vitamin D deficiency on COVID-19: a systematic review and meta-analysis of observational studies
Source: Epidemiol Health. 2021 Sep 23;43:e2021074. doi: 10.4178/epih.e2021074 (PMC8769802; doi:10.4178/epih.e2021074)
Supplement: Supplementary file 8 [file epih-43-e2021074-suppl8.docx]

Death

Severe cases

Covid-19 (+/-)

889

710

177

1589

1300

202196

| Supplementary Material 8. The distribution of samples obtained from the included studies. |
| --- |
